# The affordability of lecanemab, an amyloid-targeting therapy for Alzheimer's disease: an EADC-EC viewpoint

**DOI:** 10.1016/j.lanepe.2023.100657

**Published:** 2023-05-22

**Authors:** Linus Jönsson, Anders Wimo, Ron Handels, Gunilla Johansson, Mercè Boada, Sebastiaan Engelborghs, Lutz Frölich, Frank Jessen, Patrick Gavin Kehoe, Milica Kramberger, Alexandre de Mendonςa, Pierre Jean Ousset, Nikolaos Scarmeas, Pieter Jelle Visser, Gunhild Waldemar, Bengt Winblad

**Affiliations:** a Department of Neurobiology, Care Sciences and Society, Division of Neurogeriatrics, Karolinska Institutet, Solna, Sweden; b Department of Psychiatry and Neuropsychology, Maastricht University, Alzheimer Centre Limburg, School for Mental Health and Neurosciences, Maastricht, the Netherlands; c ACE Alzheimer Center Barcelona - International University of Catalunya, Spain & Networking Research Center on Neurodegenerative Diseases (CIBERNED), Instituto de Salud Carlos III, Madrid, Spain; d Department of Neurology and Bru-BRAIN, Center for Neurosciences, UZ Brussel & Vrije Universiteit Brussel (VUB), Brussels, Belgium; e Department of Geriatric Psychiatry, Central Institute of Mental Health, Medical Faculty Mannheim, University of Heidelberg, Germany; f Department of Psychiatry, University of Cologne, Faculty of Medicine & University Hospital Cologne, Cologne, Germany; g Department of Neurology, University Medical Centre, Ljubljana & Medical Faculty, University of Ljubljana, Slovenia; h Bristol Medical School, Institute of Medical Science, Bristol, UK; i Faculty of Medicine, University of Lisbon, Lisbon, Portugal; j Department of Internal Medicine and Clinical Gerontology, Toulouse University Hospital, Toulouse, France; k Department of Neurology, Aiginition Hospital, National and Kapodistrian University of Athens Medical School, Athens, Greece; l Department of Neurology, Columbia University, New York, NY, USA; m Department of Psychiatry and Neuropsychology, University of Maastricht & Department of Neurology, Amsterdam Centre, Amsterdam, the Netherlands; n Department of Neurology, Danish Dementia Research Centre, Copenhagen University Hospital -Rigshospitalet, Copenhagen, Denmark; o Department of Clinical Medicine, University of Copenhagen, Denmark; p Theme Inflammation and Aging, Karolinska University Hospital, Huddinge, Sweden

**Keywords:** Passive immunotherapy, Antibodies against amyloid b-peptide, Prodromal AD, Mild AD, Lecanemab, Pricing, Health economics

## Abstract

Lecanemab, an anti-amyloid antibody with effects on biomarker and clinical endpoints in early Alzheimer's Disease (AD), was granted accelerated approval by the FDA in 2023 and regulatory review in Europe is ongoing. We estimate the population potentially eligible for treatment with lecanemab in the 27 EU countries to 5.4 million individuals. Treatment costs would exceed 133 billion EUR per year if the drug is priced similarly as in the United States, amounting to over half of the total pharmaceutical expenditures in the EU. This pricing would be unsustainable; the ability to pay for high-priced therapies varies substantially across countries. Pricing similarly to what has been announced for the United States may place the drug out of reach for patients in some European countries. Disparities in access to novel amyloid-targeting agents may further deepen the inequalities across Europe in health outcomes. As representatives of the European Alzheimer's Disease Consortium Executive Committee, we call for pricing policies that allow eligible patients across Europe to access important innovations, but also continued investments in research and development. Infrastructure to follow up the usage of new therapies in routine care and new payment models may be needed to address affordability and inequalities in patient access.

## Development and regulatory assessment of amyloid-targeting therapies for Alzheimer's disease

Over the last decades, tremendous efforts have been spent on research focused on developing disease modifying treatment (DMT) for Alzheimer's disease, with impact on the underlying mechanisms causing neuronal damage, progressive dementia and subsequently loss of autonomy and death.^1^ One target for DMT is the aggregation of beta-amyloid peptide in the brain. However, there have been several failures in such drug trials.^2^ In 2021, Biogen obtained conditional accelerated approval for their drug aducanumab by the Food and Drug Administration (FDA) in the United States (US).^3^^,^^4^ Aducanumab is an antibody that reduces the amyloid burden, but the phase 3 trials did not reach consistent significant differences vs placebo. It was not approved by the European Medicines Agency (EMA) due to inconsistent effect and potentially severe side effects.^5^ A controversial factor was the very high price in the United States, 56,000 USD (52,336 EUR) per patient per year. High prices are not uncommon for specialist drugs targeted to small, well-defined patient populations, but since AD is a highly prevalent condition, Biogen's price policy was heavily criticized.^6^ The price was later reduced by half, but the sale of the drug in the US has been limited due to lack of reimbursement and resistance among dementia specialists in implementing the treatment. Result of the cost-effectiveness analysis of the Institute for Clinical and Economic Review (ICER) assigned a rating of “insufficient” for whether aducanumab provides a net health benefit for patients and estimated justifying drug prices of 2950–8360 USD per year if traditional cost-effectiveness thresholds were applied.^7^

Lecanemab is an antibody aiming at reducing the amyloid burden in the brain by targeting protofibrils, an intermediate step in amyloid plaque formation.^8^ Based on the convincing outcomes of a phase 2 trial,^8^ demonstrating reductions in brain amyloid load, quantified by positron emission tomography (PET) imaging, FDA granted lecanemab accelerated approval in January 2023.^9^ The application had earlier been granted Fast Track, Priority Review and Breakthrough Therapy designations. In addition, the recently published Clarity-AD phase 3 trial demonstrated significant effects on all clinical outcomes, including a 27% reduction in progression on the primary endpoint clinical dementia rating-sum of boxes (CDR-SB).^10^ The absolute change on the CDR-SB was −0.45 compared to placebo, which is below what some consider a clinically meaningful benefit at the individual patient level.^11^ 26.4% of patients treated with lecanemab experienced infusion-related reactions, and amyloid-related imaging abnormalities with edema or effusions (ARIA-E) were found in 12.6% of patients. A supplemental Biologics License Application has subsequently been submitted to FDA for Traditional Approval.

The regulatory application to the European Medicines Agency (EMA) has been filed and is currently under review. Given the positive phase 3 clinical trial outcomes, we believe it is likely that lecanemab will be approved in Europe later this year.

The amyloid hypothesis of AD pathogenesis has been challenged due to the large number of failures of drug development programs. However, the positive outcome of the Clarity-AD trial supports that this hypothesis is still relevant.^10^ Being the first DMT to show significant positive clinical effects in early AD, lecanemab is a great breakthrough. Lecanemab is far from being a cure for AD, but this is the first drug to show that pharmacologically targeting the underlying mechanisms of AD can lead to clinical benefits.

## The pricing and budget impact of lecanemab

Eisai announced in January 2023 that the launch price of Leqembi, the trade name for lecanemab in the US, will amount to an annual treatment cost of 26,500 USD (24,766 EUR) per patient.^12^ The pricing was substantiated by modelling an annual per-patient value to the U.S. society based on four components: a) quality-adjusted life-years (QALY) gains compared to Standard Of Care (SOC); b) willingness-to-pay (WTP) threshold; c) cost offsets compared to SOC; and d) time on treatment.^8^ The approach to pricing of lecanemab in Europe has not yet been revealed. However, for us, reference memory centers engaged in the European Alzheimer Disease Consortium (EADC, see Box 1), the pricing policy raises concerns also from a European perspective.

Box 1 **European Alzheimer's Disease Consortium (EADC).**

**Fig. 1:**
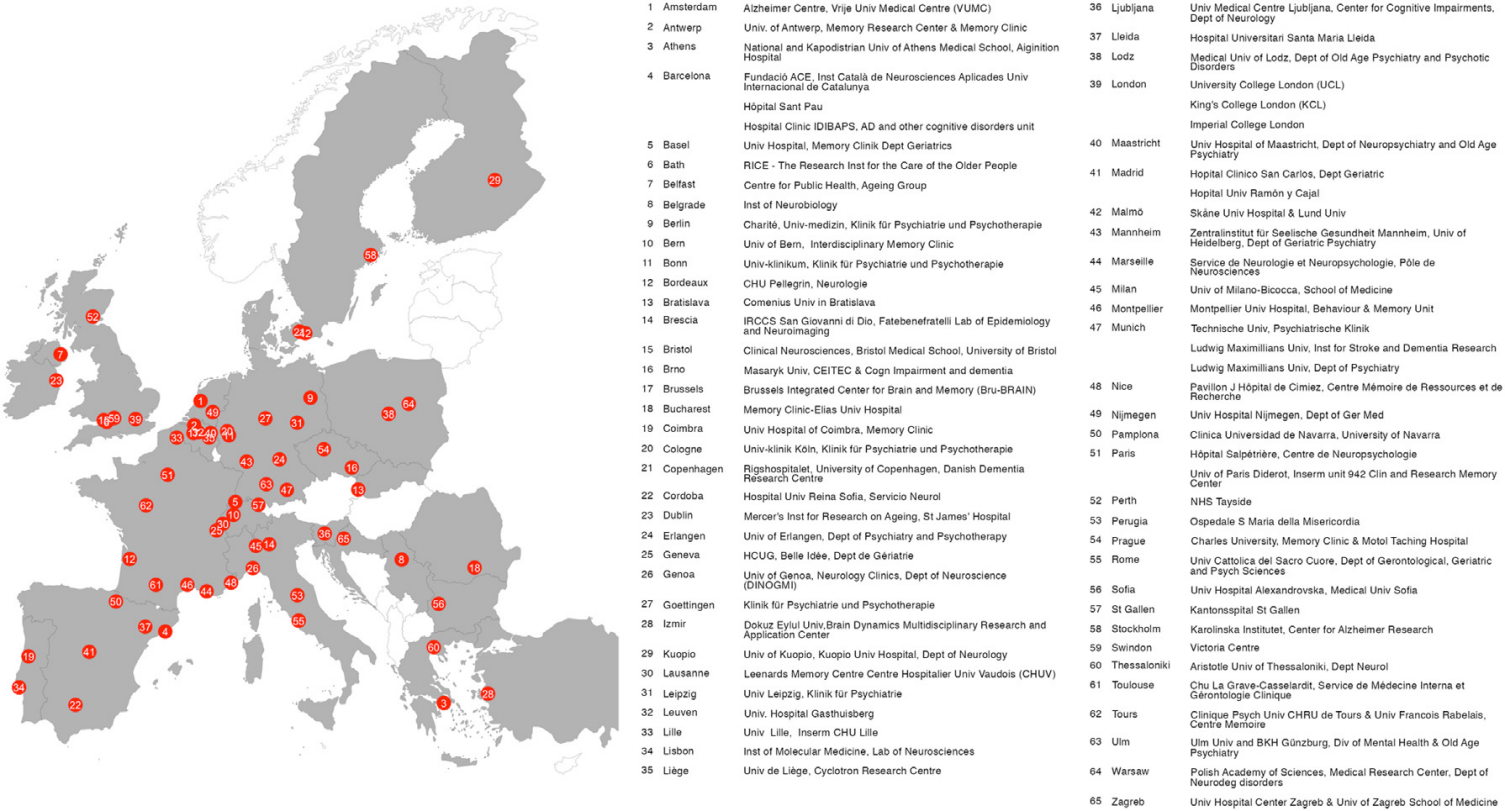
European Alzheimer's Disease Consortium (EADC) centers.

The **European Alzheimer's Disease Consortium (EADC)** is a network of 65 European specialist centres of clinical and biomedical research in AD and related dementias. The centre sites in each country are listed below and the dark grey marked countries in the map in Fig. 1 are countries holding at least one EADC centre (Website: eadc.online).

Objectives include:

• Provide an environment to promote and facilitate large European wide research studies,
• Increase the scientific understanding of AD and related dementias,
• Through consensus propose evidence based European and international research in AD,
• Impact politicians & society on AD issues, e.g. via position papers and recommendations regarding management of AD in Europe

The governance of the EADC is based upon representative democracy. The General Assembly (GA), i.e., all members taking part at meetings vote every 3rd – 4th year on which eleven members should represent the GA in an Executive Committee (EC). The following persons are members of the EC: Frank Jessen (chair), Lutz Frölich (co-chair), Milica Kramberger (coordinator), Mercè Boada, Sebastiaan Engelborghs, Patrick Kehoe, Alexandre Mendonca, Nikolaus Scarmeas, Gunhild Waldemar and Pieter Jelle Visser.

The potentially eligible patient population for treatment with lecanemab in Europe includes all patients with prodromal AD/mild cognitive impairment (MCI) due to Alzheimer's disease or mild dementia due to AD. The prevalence of prodromal AD in Europe has previously been estimated to 15.2 million,^13^ however this estimate does not take into account low detection rates (in particular in MCI) or barriers to diagnosis. We conservatively estimate the population potentially eligible for treatment in the 27 European Union (EU) countries to approximately 5.4 million individuals in 2023 (see Box 2). Treating all eligible patients in Europe with lecanemab would cost 133 billion EUR per year, assuming the drug would be priced similarly as in the US. This can be compared with the total value of the pharmaceutical market in the EU, estimated by the European federation of pharmaceutical industries and association (EFPIA) to 255 billion EUR in 2021.^14^ Thus, if used in its full potential indication, even a conservative estimate of the spending on this single drug would amount to over half of the total pharmaceutical expenditures in the EU. We believe this pricing would be unsustainable.

Box 2 **Estimated eligible number of people for treatment with lecanemab in the 27 EU countries.**

**Table:** 

Age	Amyloid-positive MCI			Mild AD dementia			Total
	Women	Men	Total	Women	Men	Total	
60–64	136,232 (60,550–282,555)	126,724 (56,322–262,837)	262,956 (116,872–545,392)	90,364 (61,415–126,604)	51,538 (34,310–72,191)	141,902 (95,725–198,795)	404,858 (212,597–744,187)
65–69	176,392 (102,121–311,009)	155,504 (90,029–274,176)	331,896 (192,150–585,185)	125,827 (85,208–176,013)	68,617 (45,052–98,684)	194,444 (130,260–274,697)	526,340 (322,410–859,882)
70–74	218,419 (151,211–319,222)	183,374 (126,950–268,005)	401,793 (278,161–587,227)	181,241 (117,172–255,434)	89,483 (58,780–127,829)	270,724 (175,952–383,263)	672,517 (454,113–970,490)
75–79	268,664 (167,518–414,059)	207,405 (129,323–319,646)	476,069 (296,841–733,705)	221,750 (145,459–314,968)	99,231 (64,619–141,181)	320,981 (210,078–456,149)	797,050 (506,919–1,189,854)
80–84	440,981 (266,252–687,814)	297,492 (179,618–464,016)	738,473 (445,870–1,151,830)	318,326 (204,567–457,219)	120,752 (78,749–175,391)	439,078 (283,316–632,610)	1,177,551 (729,186–1,784,440)
85+	752,473 (516,231–1,026,626)	371,188 (254,655–506,430)	1,123,661 (770,886–1,533,056)	527,724 (335,530–775,911)	145,232 (92,253–212,695)	672,956 (427,783–988,606)	1,796,617 (1,198,669–2,521,662)
Total	1,993,161 (1,263,883–3,041,285)	1,341,687 (836,897–2,095,110)	3,334,848 (2,100,780–5,136,395)	1,465,232 (949,351–2,106,149)	574,853 (373,763–827,971)	2,040,085 (1,323,114–2,934,120)	5,374,933 (3,423,894–8,070,515)

The estimates are based on population statistics from EuroStat (2021), combined with prevalence estimates for amyloid-positive MCI and AD dementia derived from Gustavsson et al.^13^ The proportion of patients with mild AD dementia out of all patients with AD dementia is estimated to 48% (uncertainty interval 38%–58%), based on data from the Global Burden of Disease Study 2019 [31]. Numbers are adjusted for the assumption that one third of patients with amyloid-positive MCI will be eligible for treatment. Further details on the calculations are provided in the Supplement.

Treatment with lecanemab will be given as intravenous infusions every 14 days in specialist clinics and will likely require regular monitoring with clinical follow-ups, as well as magnetic resonance imaging (MRI) investigations.^15^ A formulation for subcutaneous injection of lecanemab, currently being evaluated in clinical trials, would partly reduce the costs for administration and monitoring. Making lecanemab available to patients within the target population will require fundamental changes to the diagnostic process for patients with cognitive impairment, and considerable investments in resources for early detection, diagnostic workup, treatment facilities and monitoring including the management of side effects.^16^ The costs for identifying eligible amyloid-positive patients should be considered part of the cost of implementing a treatment strategy with amyloid-targeting therapies, along with costs and quality of life impact from adverse events. Today, many AD patients are diagnosed in later stages of dementia, and the use of specific biomarkers is limited to selected patients in specialized memory clinics. To identify and qualify a large share of eligible patients for treatment would require substantial investments in diagnostic services, potentially even targeted or general screening programs.^17^ Such investments are unlikely to be made by health care systems across Europe if the drug is seen as inaccessible for cost reasons or as offering poor cost-effectiveness. These costs may also ‘crowd out’ or displace existing patient care with established clinical utility and cost-effectiveness. Experiences from new drug introductions have demonstrated these challenges also in less common neurological indications such as multiple sclerosis.^18^

## New diagnostic pathways

Recently, important advances have been made in new diagnostic technologies that may transform our ability for early detection, fast and precise diagnosis of early AD. The development of plasma-based biomarkers may provide a useful tool in primary care, and could form part of a cost-effective diagnostic pathway for increased identification of early AD,^17^ however they require further validation before implementation in routine care.^19^ Digitalization of cognitive tests and digital tools supporting the diagnostic process may further help to increase early detection and accelerate the diagnostic process. Improving diagnostic precision at the primary care level is of critical importance to alleviate the pressure on specialist referral sites. Together, these developments raise the hope of enabling early detection and diagnosis at scale. However, without meaningful and accessible DMT (also from an affordability perspective), these developments are unlikely to be realized in practice. Currently, only a small fraction of the potentially eligible population for DMT are identified. In a study covering expert memory clinics in France, a lumbar puncture with CSF biomarker investigation was conducted in less than 10% of all patients with prodromal to mild AD in 2014.^20^ Readiness for taking up new diagnostic methods and tools is even lower in primary care, which is where patients with early AD first will have to be identified in order to reach most eligible patients.

## Assessing the cost-effectiveness of disease-modifying therapies

We also believe the pricing is likely to be unreasonable in relation to the health benefits that have so far been demonstrated. The justification of the announced US price is based on a health economic evaluation which has recently been published.^21^^,^^22^ No estimates of cost-effectiveness have yet been presented for Europe to our knowledge, but some observations can be made from the disclosed results for the US. Treatment with lecanemab was projected in a simulation model to delay disease progression by nearly 3 years on average.^12^ The duration of the clinical trial from which efficacy data for lecanemab were obtained was 18 months, thus it is clear that the effects are extrapolated far beyond the period of data availability. Further, a 3-year delay resulting from a 3.6 year treatment duration (as estimated by the company^12^) appears to indicate a higher treatment effect than the 27% reduction in disease progression demonstrated in the trial. The effect on CDR-SB in Clarity-AD is consistent with a time delay in disease progression of approximately 6 months. Extrapolations beyond the period of data availability are associated with considerable uncertainty. It would be reasonable to base pricing on demonstrated clinical benefits rather than extrapolations. The uncertainty around the long-term clinical benefits needs to be taken into account when assessing the value of the therapy. The cost-effectiveness of lecanemab needs to be assessed by independent investigators (without direct support or influence from manufacturers, payers or HTA agencies), with careful consideration of the uncertainty in the underlying clinical and economic data, and the methods and results published transparently and in detail.

The willingness to pay per QALY employed by Eisai was 200,000 USD (187,000 EUR) per QALY. This might be an overestimate for the US market, and certainly for many European countries. The National Institute for Health and Clinical Excellence (NICE) in the United Kingdom has since long used a threshold value of 20–30,000 GBP (23,000–34,000 EUR) per QALY.^23^ If the cost-per-QALY threshold is set to 34,000 EUR in the above analysis, the maximum yearly cost of treatment would decrease to 8104 EUR^q^ (including diagnosis, administration, monitoring and all other treatment-associated costs).

Other countries, e.g., Germany, do not rely on a cost per QALY threshold for reimbursement decisions. Further, it is not obvious that a drug with such a wide target patient population should be priced at the maximum willingness to pay. At this price, from a societal perspective the introduction of the drug generates no net value, as the entire added value of the drug (health benefits and cost offsets) is claimed by the manufacturer through the drug cost (unless the profits of the manufacturer are included in societal gains). This may not be reasonable for a drug which is based on decades of basic and applied research, much of it publicly funded. It can be argued that the share of the value captured by the manufacturer should be lower in this circumstance than for products with a narrower target patient population.^24^

To improve cost-effectiveness in clinical use, reimbursement for lecanemab could be restricted to subgroups of patients within the overall target indication. ApoE4 noncarrier status, male sex and high age were among factors observed to be associated with higher clinical benefits.^10^ Limiting reimbursement to subgroups would have the combined effect of reducing the cost per QALY ratio as well as the budget impact.

The ability to pay high acquisition costs for novel therapies varies substantially across European countries. Due to the possibility of parallel trade, it is not possible to maintain large differences in official list prices for drugs between countries. Thus, a price similarly to what has been announced for the United States is likely to place the drug out of reach for patients in some European countries and regions.^25^ The practice of confidential discounts on list prices has become more widespread as an attempt to differentiate pricing across countries. However, the secretive nature of these agreements leads to uncertainty about the true price actually paid for the drug and thus the actual cost-effectiveness in clinical use,^26^ and may undermine trust in the decision making process.^27^

## Consequences for inequalities in access and health

Disparities in access to novel amyloid-targeting agents may further deepen the inequalities across Europe in health outcomes. The care structure for persons with dementia and the resource utilization and costs of dementia care differs substantially across Europe, both between and within countries and between majority and ethnic minority populations.^28^ Simplifying, Northern European countries tend to have higher availability of formal care services such as home help and nursing homes, while in Southern Europe family caregivers provide most of the care.^29^ Thus, the opportunity for offsetting high drug costs with lower costs for formal care differ substantially across countries, and the evidence for such offsets currently comes only from model projections. There is also a high potential value in offsetting informal care costs. But as these do not appear on health care budgets, there may be less willingness to support high drug costs against such offsets. As a consequence, the introduction of novel AD therapies may have the undesired effect of decreasing rather than improving health equity, a stated objective by the European Union, the WHO and within the UN sustainable development goals.^30^

## Conclusions

As representatives of the EADC Executive Committee, we call for pricing policies that will allow eligible patients across Europe to access important innovations, as well as continued investments in research, development, and clinical implementation of new therapies. The uncertainty around the long-term clinical and economic value of amyloid-targeted therapies highlights the need for an infrastructure to follow up the usage of these drugs and their impact in routine care. Further, there may be a need for alternative contracting and payment models to address issues related to affordability and inequalities in patient access.

## Process for developing the viewpoint manuscript

BW first proposed the development of a manuscript on the topic of affordability and economic impact of lecanemab to the EC on January 20, 2023. The EC approved the proposal, and AW and LJ were appointed to conduct the statistical analyses and draft the first version of the manuscript. The first draft was circulated on February 15 among all EC members who provided written feedback via email. The feedback was consolidated and key points discussed among a smaller working group of authors (AW, LJ, GJ and BW). A revised version was circulated with EC members on February 27, and after minor corrections it was approved by all committee members. Following reviewer comments revised versions of the manuscript was circulated for approval among co-authors. The paper was presented by AW at the EADC General Assembly meeting in Toulouse on April 28. No systematic literature review was conducted in support of the manuscript as there is currently limited literature on the health economic impact of lecanemab.

## Contributors

The views and conclusions expressed in this Viewpoint article were developed through discussions and written exchanges including all members of the EADC Executive Committee. These exchanges took place during the period January–March 2023.

LJ: Methodology, Formal analysis, Writing - Original Draft, Visualization

AW: Conceptualization, Methodology, Formal analysis, Writing - Original Draft

GJ: Writing - Reviewing & Editing, Project, Administration, Visualization

RH, MB, SE, LF, FJ, PK, MK, AM, PO, NS, PV, GW: Writing - Reviewing & Editing

BW: Conceptualization, Writing - Reviewing & Editing, Supervision

## Ethics committee approval

Not applicable.

## Declaration of interests

No specific funding was received for the preparation of this manuscript. Authors have completed separate CoI forms.

The authors declare the following Conflicts of Interest:

LJ received research grants (paid to institution) by Vinnova, FORTE and Novo Nordisk, license fees for the RUD instrument paid to European Health Economics, advisory board honorarium from Laboratoires Servier, travel support from BioArctic AB.

AW received grants from Vinnova and EU (PREDEM, MOPEAD, PRODEMOS, EURO-FINGER, PROMINENT, PMI-AD, ADDITION, paid to institution), and is a license holder of the RUD instrument. Member of MSAP for ADI (un-paid).

RH received research grants from JPND, ZonMW, IMI, H2020 (paid to institution), consulting fees from Lilly Nederland, iMTA, Biogen Nederlands, Biogen MA Inc, Eisai Inc (paid to institution), member of ISPOR modeling SIG and IPECAD modeling group (un-paid).

MB is Member of the Advisory Boards Grifols, Roche, Lilly, Araclon Biotech, Merck, Zambon, Biogen, Novo- Nordisk, Bioiberica, Eisai, Servier, Schwabe Pharma.

SE received research grants from Interreg Vlaanderen-Nederland, Research Foundation Flanders (FWO), VLAIO, GSKE/FMRE (paid to institution), consulting fees from Icometrix, Novartis, Eisai (paid to institution), personal consulting fees from Roche and Biogen, personal honoraria from Eisai, Roche, travel support to institution from Biogen, Patent EP3452830B1 (institution), member of SMB/SAB for EU-H2020 project RECAGE. VP of Belgian Dementia Council (unpaid).

LF received grants from EU (RADAR-AD, RECAGE, FRAILBRAIN, paid to institution), personal consulting fees from Biogen, Eisai, Grifols, Hummingbird, Infecto-Pharm, Janssen-Cilag, MSD, Neurimmune, Functional Neuromodulation, Noselab, NovoNordisk, Roche, TauRX, Schwabe, lecture honoraria from Hoffmann-LaRoche and Schwabe, personal advisory board honorarium from Avanir/Otsuka, Pharmatropix, FZ Jülich, Charité Berlin, Neuroscios, reMYND, Vivoryon.

FJ received personal consulting fees and lecture honoraria from Eisai, Biogen, Lilly, and personal honoraria for participation in advisory board from AC Immune.

PJO received grants from Alector, Alzheon, Araclon Biotech, Biogen, Avanir Pharmaceuticals, Cortexyme, Eisai, Eli Lilly, Green Valley Pharmaceutical, Hoffman-La Roche, Janssen, Novartis Pharmaceuticals, NovoNordisk, TauRx, UCB Biopharma (paid to institution).

NS received grants from EU (EPAD) and NovoNordisk (paid to institution). Personal fee as member of SAB from Albert Einstein College of Medicine – NIH funded.

PJV received grants from EU (AMYPAD, RADAR-AD, EPND), Zon-MW, Biogen, Amyloid biomarker study (paid to institution), honoraria from workshop grant writing by Stiftung Synapsis, Alzheimer Forschung Schweiz. Patent holder (PCT/NL2020/050216) on AD subtypes (all paid to institution).

GW received research grant from Danish Ministry of Health (paid to institution).

BW received personal honorarium for a 2 h meeting with BioArctic, member of SMB for Alzinova, member of SAB for Resverlogix, stocks in AlzeCure pharma.

GJ, PK, MK and AM declare no conflicts of interest.

## Footnotes

q ((0.64 ∗ 34000) + 7415)/3.6 ≈ 8104.
