# Supplementary material for: The affordability of lecanemab, an amyloid-targeting therapy for Alzheimer's disease: an EADC-EC viewpoint
Source: Lancet Reg Health Eur. 2023 May 22;29:100657. doi: 10.1016/j.lanepe.2023.100657 (PMC10220264; doi:10.1016/j.lanepe.2023.100657)
Supplement: Supplementary Tables S1–S5 [file mmc1.docx]

Supplement: calculation of potential number of eligible patients for anti-amyloid therapy in EU-27

2023-02-22

## 1. Population by age group, sex and country

Demographic estimates were obtained from EuroStat (table demo_pjangroup) for 2021.

Table 1. Population by country, age and sex ('000)

|  | Women | | | | | | Men | | | | | |  |
| --- | --- | --- | --- | --- | --- | --- | --- | --- | --- | --- | --- | --- | --- |
| Country | 60-64 | 65-69 | 70-74 | 75-79 | 80-84 | 85+ | 60-64 | 65-69 | 70-74 | 75-79 | 80-84 | 85+ | All |
| Austria | 300 | 242 | 222 | 192 | 162 | 150 | 288 | 218 | 189 | 150 | 116 | 74 | 2,303 |
| Belgium | 372 | 326 | 296 | 213 | 185 | 221 | 363 | 306 | 265 | 174 | 131 | 111 | 2,963 |
| Bulgaria | 247 | 251 | 252 | 178 | 124 | 98 | 221 | 200 | 176 | 110 | 67 | 47 | 1,971 |
| Croatia | 153 | 144 | 125 | 91 | 82 | 67 | 139 | 127 | 94 | 59 | 46 | 28 | 1,155 |
| Cyprus | 26 | 23 | 21 | 15 | 11 | 9 | 25 | 22 | 19 | 13 | 9 | 6 | 199 |
| Czech Republic | 321 | 359 | 347 | 247 | 154 | 141 | 305 | 313 | 274 | 170 | 90 | 62 | 2,783 |
| Denmark | 175 | 163 | 165 | 137 | 87 | 82 | 173 | 156 | 153 | 120 | 68 | 45 | 1,524 |
| Estonia | 47 | 46 | 41 | 31 | 31 | 28 | 39 | 33 | 25 | 16 | 12 | 8 | 357 |
| Finland | 182 | 184 | 188 | 129 | 95 | 105 | 175 | 170 | 166 | 104 | 65 | 50 | 1,613 |
| France | 2,166 | 2,065 | 1,983 | 1,266 | 1,091 | 1,551 | 1,977 | 1,820 | 1,709 | 1,016 | 765 | 731 | 18,140 |
| Germany | 2,952 | 2,565 | 2,114 | 1,917 | 1,998 | 1,655 | 2,867 | 2,334 | 1,855 | 1,551 | 1,432 | 851 | 24,091 |
| Greece | 367 | 327 | 309 | 246 | 227 | 229 | 321 | 288 | 261 | 200 | 162 | 158 | 3,095 |
| Hungary | 334 | 374 | 299 | 235 | 167 | 145 | 279 | 283 | 204 | 138 | 78 | 55 | 2,591 |
| Ireland | 134 | 116 | 99 | 74 | 50 | 53 | 130 | 113 | 95 | 68 | 41 | 32 | 1,005 |
| Italy | 2,045 | 1,821 | 1,832 | 1,407 | 1,325 | 1,464 | 1,901 | 1,653 | 1,610 | 1,141 | 953 | 737 | 17,889 |
| Latvia | 75 | 67 | 60 | 51 | 48 | 37 | 58 | 45 | 34 | 24 | 18 | 10 | 527 |
| Lithuania | 115 | 93 | 82 | 73 | 64 | 57 | 89 | 63 | 47 | 34 | 26 | 18 | 761 |
| Luxembourg | 17 | 14 | 12 | 9 | 7 | 8 | 18 | 14 | 11 | 8 | 5 | 4 | 127 |
| Malta | 16 | 14 | 15 | 10 | 7 | 7 | 16 | 14 | 14 | 9 | 5 | 3 | 130 |
| Netherlands | 571 | 508 | 497 | 340 | 253 | 254 | 567 | 496 | 474 | 304 | 196 | 135 | 4,595 |
| Poland | 1,407 | 1,374 | 1,103 | 621 | 571 | 586 | 1,247 | 1,114 | 806 | 389 | 292 | 228 | 9,738 |
| Portugal | 368 | 341 | 309 | 257 | 212 | 225 | 319 | 286 | 248 | 187 | 136 | 109 | 2,997 |
| Romania | 654 | 677 | 557 | 371 | 333 | 273 | 574 | 537 | 404 | 235 | 181 | 138 | 4,934 |
| Slovakia | 186 | 184 | 144 | 101 | 70 | 61 | 168 | 151 | 104 | 60 | 34 | 24 | 1,287 |
| Slovenia | 72 | 70 | 58 | 43 | 37 | 39 | 72 | 66 | 50 | 32 | 24 | 16 | 579 |
| Spain | 1,552 | 1,303 | 1,190 | 990 | 774 | 1,037 | 1,465 | 1,188 | 1,025 | 791 | 534 | 539 | 12,388 |
| Sweden | 284 | 272 | 282 | 238 | 155 | 168 | 285 | 265 | 268 | 219 | 126 | 95 | 2,657 |
| Total | 15,138 | 13,923 | 12,602 | 9,482 | 8,320 | 8,750 | 14,081 | 12,275 | 10,580 | 7,322 | 5,612 | 4,314 | 122,399 |

## 2. Prevalence of mild AD dementia

Gustavsson et al present prevalence estimates for amyloid-positive dementia by age, sex and region. Source: Gustavsson (2022) The prevalence estimates above are for all stages of dementia (mild, moderate, severe). To obtain an estimate of the number of patients with mild AD dementia, we assume a proportion of mild cases out of all AD dementia cases of 48%. This is based on data from the Global Burden of Illness Study, obtained through the Global Health Data Exchange (<https://vizhub.healthdata.org/gbd-results/>).

Table 2. Prevalence of amyloid-positive dementia

|  | Women | | | | | | Men | | | | | |
| --- | --- | --- | --- | --- | --- | --- | --- | --- | --- | --- | --- | --- |
| Country | 60-64 | 65-69 | 70-74 | 75-79 | 80-84 | 85+ | 60-64 | 65-69 | 70-74 | 75-79 | 80-84 | 85+ |
| Austria | 0.013 | 0.020 | 0.032 | 0.052 | 0.085 | 0.133 | 0.007 | 0.011 | 0.017 | 0.028 | 0.045 | 0.071 |
| Belgium | 0.013 | 0.020 | 0.032 | 0.052 | 0.085 | 0.133 | 0.007 | 0.011 | 0.017 | 0.028 | 0.045 | 0.071 |
| Bulgaria | 0.011 | 0.016 | 0.024 | 0.037 | 0.058 | 0.087 | 0.010 | 0.014 | 0.020 | 0.029 | 0.042 | 0.060 |
| Croatia | 0.011 | 0.016 | 0.024 | 0.037 | 0.058 | 0.087 | 0.010 | 0.014 | 0.020 | 0.029 | 0.042 | 0.060 |
| Cyprus | 0.013 | 0.020 | 0.032 | 0.052 | 0.085 | 0.133 | 0.007 | 0.011 | 0.017 | 0.028 | 0.045 | 0.071 |
| Czech Republic | 0.011 | 0.016 | 0.024 | 0.037 | 0.058 | 0.087 | 0.010 | 0.014 | 0.020 | 0.029 | 0.042 | 0.060 |
| Denmark | 0.013 | 0.020 | 0.032 | 0.052 | 0.085 | 0.133 | 0.007 | 0.011 | 0.017 | 0.028 | 0.045 | 0.071 |
| Estonia | 0.006 | 0.008 | 0.019 | 0.035 | 0.068 | 0.138 | 0.006 | 0.008 | 0.019 | 0.035 | 0.068 | 0.138 |
| Finland | 0.013 | 0.020 | 0.032 | 0.052 | 0.085 | 0.133 | 0.007 | 0.011 | 0.017 | 0.028 | 0.045 | 0.071 |
| France | 0.013 | 0.020 | 0.032 | 0.052 | 0.085 | 0.133 | 0.007 | 0.011 | 0.017 | 0.028 | 0.045 | 0.071 |
| Germany | 0.013 | 0.020 | 0.032 | 0.052 | 0.085 | 0.133 | 0.007 | 0.011 | 0.017 | 0.028 | 0.045 | 0.071 |
| Greece | 0.013 | 0.020 | 0.032 | 0.052 | 0.085 | 0.133 | 0.007 | 0.011 | 0.017 | 0.028 | 0.045 | 0.071 |
| Hungary | 0.011 | 0.016 | 0.024 | 0.037 | 0.058 | 0.087 | 0.010 | 0.014 | 0.020 | 0.029 | 0.042 | 0.060 |
| Ireland | 0.013 | 0.020 | 0.032 | 0.052 | 0.085 | 0.133 | 0.007 | 0.011 | 0.017 | 0.028 | 0.045 | 0.071 |
| Italy | 0.013 | 0.020 | 0.032 | 0.052 | 0.085 | 0.133 | 0.007 | 0.011 | 0.017 | 0.028 | 0.045 | 0.071 |
| Latvia | 0.006 | 0.008 | 0.019 | 0.035 | 0.068 | 0.138 | 0.006 | 0.008 | 0.019 | 0.035 | 0.068 | 0.138 |
| Lithuania | 0.006 | 0.008 | 0.019 | 0.035 | 0.068 | 0.138 | 0.006 | 0.008 | 0.019 | 0.035 | 0.068 | 0.138 |
| Luxembourg | 0.013 | 0.020 | 0.032 | 0.052 | 0.085 | 0.133 | 0.007 | 0.011 | 0.017 | 0.028 | 0.045 | 0.071 |
| Malta | 0.006 | 0.008 | 0.019 | 0.035 | 0.068 | 0.138 | 0.006 | 0.008 | 0.019 | 0.035 | 0.068 | 0.138 |
| Netherlands | 0.013 | 0.020 | 0.032 | 0.052 | 0.085 | 0.133 | 0.007 | 0.011 | 0.017 | 0.028 | 0.045 | 0.071 |
| Poland | 0.011 | 0.016 | 0.024 | 0.037 | 0.058 | 0.087 | 0.010 | 0.014 | 0.020 | 0.029 | 0.042 | 0.060 |
| Portugal | 0.013 | 0.020 | 0.032 | 0.052 | 0.085 | 0.133 | 0.007 | 0.011 | 0.017 | 0.028 | 0.045 | 0.071 |
| Romania | 0.011 | 0.016 | 0.024 | 0.037 | 0.058 | 0.087 | 0.010 | 0.014 | 0.020 | 0.029 | 0.042 | 0.060 |
| Slovakia | 0.011 | 0.016 | 0.024 | 0.037 | 0.058 | 0.087 | 0.010 | 0.014 | 0.020 | 0.029 | 0.042 | 0.060 |
| Slovenia | 0.011 | 0.016 | 0.024 | 0.037 | 0.058 | 0.087 | 0.010 | 0.014 | 0.020 | 0.029 | 0.042 | 0.060 |
| Spain | 0.013 | 0.020 | 0.032 | 0.052 | 0.085 | 0.133 | 0.007 | 0.011 | 0.017 | 0.028 | 0.045 | 0.071 |
| Sweden | 0.013 | 0.020 | 0.032 | 0.052 | 0.085 | 0.133 | 0.007 | 0.011 | 0.017 | 0.028 | 0.045 | 0.071 |

Table 3. Number of patients with amyloid-positive mild AD dementia ('000)

|  | Women | | | | | | Men | | | | | |  |
| --- | --- | --- | --- | --- | --- | --- | --- | --- | --- | --- | --- | --- | --- |
| Country | 60-64 | 65-69 | 70-74 | 75-79 | 80-84 | 85+ | 60-64 | 65-69 | 70-74 | 75-79 | 80-84 | 85+ | All |
| Austria | 2 | 2 | 3 | 5 | 7 | 10 | 1 | 1 | 2 | 2 | 3 | 3 | 39 |
| Belgium | 2 | 3 | 5 | 5 | 8 | 14 | 1 | 2 | 2 | 2 | 3 | 4 | 51 |
| Bulgaria | 1 | 2 | 3 | 3 | 3 | 4 | 1 | 1 | 2 | 2 | 1 | 1 | 25 |
| Croatia | 1 | 1 | 1 | 2 | 2 | 3 | 1 | 1 | 1 | 1 | 1 | 1 | 15 |
| Cyprus | 0 | 0 | 0 | 0 | 0 | 1 | 0 | 0 | 0 | 0 | 0 | 0 | 3 |
| Czech Republic | 2 | 3 | 4 | 4 | 4 | 6 | 1 | 2 | 3 | 2 | 2 | 2 | 35 |
| Denmark | 1 | 2 | 3 | 3 | 4 | 5 | 1 | 1 | 1 | 2 | 1 | 2 | 25 |
| Estonia | 0 | 0 | 0 | 1 | 1 | 2 | 0 | 0 | 0 | 0 | 0 | 1 | 6 |
| Finland | 1 | 2 | 3 | 3 | 4 | 7 | 1 | 1 | 1 | 1 | 1 | 2 | 27 |
| France | 14 | 20 | 30 | 32 | 45 | 99 | 7 | 10 | 14 | 14 | 17 | 25 | 324 |
| Germany | 18 | 25 | 32 | 48 | 82 | 106 | 10 | 12 | 15 | 21 | 31 | 29 | 428 |
| Greece | 2 | 3 | 5 | 6 | 9 | 15 | 1 | 2 | 2 | 3 | 3 | 5 | 56 |
| Hungary | 2 | 3 | 3 | 4 | 5 | 6 | 1 | 2 | 2 | 2 | 2 | 2 | 33 |
| Ireland | 1 | 1 | 2 | 2 | 2 | 3 | 0 | 1 | 1 | 1 | 1 | 1 | 15 |
| Italy | 13 | 17 | 28 | 35 | 54 | 93 | 6 | 9 | 13 | 15 | 21 | 25 | 330 |
| Latvia | 0 | 0 | 1 | 1 | 2 | 2 | 0 | 0 | 0 | 0 | 1 | 1 | 8 |
| Lithuania | 0 | 0 | 1 | 1 | 2 | 4 | 0 | 0 | 0 | 1 | 1 | 1 | 12 |
| Luxembourg | 0 | 0 | 0 | 0 | 0 | 1 | 0 | 0 | 0 | 0 | 0 | 0 | 2 |
| Malta | 0 | 0 | 0 | 0 | 0 | 0 | 0 | 0 | 0 | 0 | 0 | 0 | 2 |
| Netherlands | 4 | 5 | 8 | 8 | 10 | 16 | 2 | 3 | 4 | 4 | 4 | 5 | 72 |
| Poland | 7 | 11 | 13 | 11 | 16 | 24 | 6 | 7 | 8 | 5 | 6 | 7 | 121 |
| Portugal | 2 | 3 | 5 | 6 | 9 | 14 | 1 | 2 | 2 | 3 | 3 | 4 | 53 |
| Romania | 3 | 5 | 6 | 7 | 9 | 11 | 3 | 4 | 4 | 3 | 4 | 4 | 63 |
| Slovakia | 1 | 1 | 2 | 2 | 2 | 3 | 1 | 1 | 1 | 1 | 1 | 1 | 15 |
| Slovenia | 0 | 1 | 1 | 1 | 1 | 2 | 0 | 0 | 0 | 0 | 0 | 0 | 8 |
| Spain | 10 | 13 | 18 | 25 | 32 | 66 | 5 | 6 | 8 | 11 | 12 | 18 | 223 |
| Sweden | 2 | 3 | 4 | 6 | 6 | 11 | 1 | 1 | 2 | 3 | 3 | 3 | 45 |
| Total | 90 | 126 | 181 | 222 | 318 | 528 | 52 | 69 | 89 | 99 | 121 | 145 | 2,040 |

## 3. Prevalence of amyloid-positive MCI

Gustavsson et al present prevalence estimates for amyloid-positive dementia by age, sex and region.Source: Gustavsson (2022)

Table 4. Prevalence of amyloid-positive MCI by age

| Age | Prevalence |
| --- | --- |
| 60-64 | 0.027 |
| 65-69 | 0.038 |
| 70-74 | 0.052 |
| 75-79 | 0.085 |
| 80-84 | 0.159 |
| 85+ | 0.258 |

Far from all patients with MCI will ever seek medical care. Some may improve without treatment, others may die from other causes before the cognitive symptoms come to clinical attention. We conservatively assume that only 1 in 3 amyloid-positive MCI patients will be eligible for therapy. Thus, the prevalence estimates for amyloid-positive MCI presented above are divided by 3 as the number of eligible patients is calculated. The estimated eligible patients per country, age and sex is presented in table 5 below.

Table 5. Number of patients with amyloid-positive MCI ('000)

|  | Women | | | | | | Men | | | | | |  |
| --- | --- | --- | --- | --- | --- | --- | --- | --- | --- | --- | --- | --- | --- |
| Country | 60-64 | 65-69 | 70-74 | 75-79 | 80-84 | 85+ | 60-64 | 65-69 | 70-74 | 75-79 | 80-84 | 85+ | All |
| Austria | 3 | 3 | 4 | 5 | 9 | 13 | 3 | 3 | 3 | 4 | 6 | 6 | 62 |
| Belgium | 3 | 4 | 5 | 6 | 10 | 19 | 3 | 4 | 5 | 5 | 7 | 10 | 81 |
| Bulgaria | 2 | 3 | 4 | 5 | 7 | 8 | 2 | 3 | 3 | 3 | 4 | 4 | 48 |
| Croatia | 1 | 2 | 2 | 3 | 4 | 6 | 1 | 2 | 2 | 2 | 2 | 2 | 29 |
| Cyprus | 0 | 0 | 0 | 0 | 1 | 1 | 0 | 0 | 0 | 0 | 0 | 0 | 5 |
| Czech Republic | 3 | 5 | 6 | 7 | 8 | 12 | 3 | 4 | 5 | 5 | 5 | 5 | 67 |
| Denmark | 2 | 2 | 3 | 4 | 5 | 7 | 2 | 2 | 3 | 3 | 4 | 4 | 39 |
| Estonia | 0 | 1 | 1 | 1 | 2 | 2 | 0 | 0 | 0 | 0 | 1 | 1 | 10 |
| Finland | 2 | 2 | 3 | 4 | 5 | 9 | 2 | 2 | 3 | 3 | 3 | 4 | 42 |
| France | 19 | 26 | 34 | 36 | 58 | 133 | 18 | 23 | 30 | 29 | 41 | 63 | 510 |
| Germany | 27 | 32 | 37 | 54 | 106 | 142 | 26 | 30 | 32 | 44 | 76 | 73 | 679 |
| Greece | 3 | 4 | 5 | 7 | 12 | 20 | 3 | 4 | 5 | 6 | 9 | 14 | 90 |
| Hungary | 3 | 5 | 5 | 7 | 9 | 12 | 3 | 4 | 4 | 4 | 4 | 5 | 63 |
| Ireland | 1 | 1 | 2 | 2 | 3 | 5 | 1 | 1 | 2 | 2 | 2 | 3 | 25 |
| Italy | 18 | 23 | 32 | 40 | 70 | 126 | 17 | 21 | 28 | 32 | 51 | 63 | 521 |
| Latvia | 1 | 1 | 1 | 1 | 3 | 3 | 1 | 1 | 1 | 1 | 1 | 1 | 14 |
| Lithuania | 1 | 1 | 1 | 2 | 3 | 5 | 1 | 1 | 1 | 1 | 1 | 2 | 20 |
| Luxembourg | 0 | 0 | 0 | 0 | 0 | 1 | 0 | 0 | 0 | 0 | 0 | 0 | 3 |
| Malta | 0 | 0 | 0 | 0 | 0 | 1 | 0 | 0 | 0 | 0 | 0 | 0 | 3 |
| Netherlands | 5 | 6 | 9 | 10 | 13 | 22 | 5 | 6 | 8 | 9 | 10 | 12 | 115 |
| Poland | 13 | 17 | 19 | 18 | 30 | 50 | 11 | 14 | 14 | 11 | 15 | 20 | 233 |
| Portugal | 3 | 4 | 5 | 7 | 11 | 19 | 3 | 4 | 4 | 5 | 7 | 9 | 83 |
| Romania | 6 | 9 | 10 | 11 | 18 | 24 | 5 | 7 | 7 | 7 | 10 | 12 | 123 |
| Slovakia | 2 | 2 | 2 | 3 | 4 | 5 | 2 | 2 | 2 | 2 | 2 | 2 | 29 |
| Slovenia | 1 | 1 | 1 | 1 | 2 | 3 | 1 | 1 | 1 | 1 | 1 | 1 | 15 |
| Spain | 14 | 17 | 21 | 28 | 41 | 89 | 13 | 15 | 18 | 22 | 28 | 46 | 352 |
| Sweden | 3 | 3 | 5 | 7 | 8 | 14 | 3 | 3 | 5 | 6 | 7 | 8 | 72 |
| Total | 136 | 176 | 218 | 269 | 441 | 752 | 127 | 156 | 183 | 207 | 297 | 371 | 3,335 |
